# The National Veterinary and Sanitary Association

**Published:** 1887-01

**Authors:** 


### EDITORIAL DEPARTMENT.

*Conventions in Chicago.—National Veterinary and Sanitary Association of the United States. National Cattle Growers' Association.*

The third week of October was marked by the meeting of these two associations in Chicago and great good should be the result of them.

### THE NATIONAL VETERINARY AND SANITARY ASSOCIATION

Met on October 15th, and continued its sessions for three days. But little time was used in adopting a simple constitution, and effecting a permament organization.

After a short address by the president, in which he outlined the object of the meeting, the association went at once energetically to work on the most important question of the moment, Contagious Pleuro-pneumonia.

Letters relating to the subject and regretting absence, from Prof. A. Liautard, of New York, Prof. C. A. Lyman, of Boston, Dr. Holcombe, State Veterinarian of Kansas, and others, were read.

Dr. Gadsden, Dr. Salmon, and a number of other gentlemen who had been inspecting the cattle stables of Chicago, read papers or made addresses defining the exact status of the extensive outbreak of the lung plague in the neighborhood, and expressed their views in regard to the means to be employed for its suppression. Dr. Gadsden showed some specimens of diseased lungs, which he had removed the day before, from cattle in the Chicago stables. All discussion of papers was to the point; there was but little dissention among the professional members, while the lay members of the association only asked for definite information, and for the reasons of the danger, which they appreciated was threatening their herds. The following resolutions were adopted:

WHEREAS, The existence of contagious diseases among animals in the United States is annually a source of great loss; and

WHEREAS, The great cattle industry, commerce and food supply of the country are particularly threatened by the recent extensions of contagious pleuro-pneumonia; and

WHEREAS, Real and alleged outbreaks of contagious disease require constant investigation and control; therefore, be it

*Resolved,* The veterinarians and veterinary sanitary boards of the United States in convention assembled recognize the wisdom of Congress in establishing the Bureau of Animal Industry, and, while heartily appreciating the valuable services rendered by this bureau in the past, we would recommend such additional legislation as to enable it to effectually extirpate contagious pleuro-pneumonia, and to control such other contagious diseases as may from time to time appear; and

WHEREAS, Contagious pleuro-pneumonia of cattle exists in certain restricted localities of the United States; and

WHEREAS, Inoculation is being practiced in certain States as a preventive measure, and is being advocated for general adoption; and

WHEREAS, The experience of other nations has shown that this contagion is prevalent in localities where inoculation is practiced, and that inoculated cattle are dangerous to other animals with which they afterwards cohabit; and

WHEREAS, The veterinary profession of Europe condemns inoculation, except in localities that are thoroughly infected and where no effort is being made to extirpate the plague; therefore, be it

*Resolved,* That considering the limited territory infected in this country, every effort should be directed to the thorough eradication of this disease from America.

*Resolved,* That we consider inoculation to be an extremely dangerous and objectionable practice in the present condition of affairs in this country, and one which should be discouraged by the veterinary profession and prohibited by law as long as there is a possibility of stamping out the disease.

The association was unanimous in its expression, that extirpation, the pole-axe, is the only reasonable treatment and method of handling this disease, whatever the cost.

While the attendance at the meetings was smaller than had been hoped for, it was representative of the West, but two or three of the state and territorial veterinarians being absent, and those who were present were supported by members of their live stock commissions. The East was unfortunately but poorly represented in numbers.

The usefulness of the Bureau of Animal Industry, even with its crippled means, was happily demonstrated by the presence of Dr. Salmon, with two or three of his assistants, who were able to furnish any desired information, in regard to infected districts and the veterinary force in charge of such regions, notwithstanding the absence of the local delegates.

The following officers were chosen for the ensuing year, and the place and time of the next meeting was fixed, at the call of the President, to coincide with that of the National Cattle Growers' Association: The Hon. J. L. Brush, Greeley, Colo., President; Dr. J. D. Hopkins, Cheyenne, Wyoming, and Dr. V. T. Atkinson, Wisconsin, Vice-Presidents; Dr. Paul Paquin, Columbia, Missouri, Secretary.

